# Optimal design of Halbach magnetized magnetic screw for wave energy converters based on KELM network optimized by weighted mean of vectors algorithm

**DOI:** 10.1371/journal.pone.0329295

**Published:** 2025-08-14

**Authors:** Qiongfang Zhang, Haitao Yu, Yulei Liu, Qinghai Qin, Jiahui Zhang

**Affiliations:** School of Electrical Engineering, Southeast University, Nanjing, Jiangsu, China; inSync Mirror LLC, UNITED STATES OF AMERICA

## Abstract

This paper investigates the parameter optimization problem of Halbach magnetized magnetic screw (HMMS) for wave energy converter (WECs). A magnetic screw with improved Halbach magnetized PMs arrays is presented. To further enhance the thrust density of HMMS, a HMMS optimization model is proposed based on the kernel extreme learning machine (KELM) optimized by weIght meaN oF vectOrs (INFO) algorithm. The topology and working principle of HMMS are introduced. Based on comprehensive sensitivity analysis, the design space is stratified, and the nonlinear coupling relationships between parameters are addressed using the Kriging model to enhance the accuracy and efficiency of the optimization process. The INFO algorithm optimizes kernel parameters and regularization coefficients of KELM, which critically affect its output accuracy. By establishing the INFO-KELM optimization model, the final optimized structure is obtained. The FEA is utilized to assess the properties of the HMMS. Compared with the traditional radially magnetized screw, the thrust force of the optimized HMMS increased by 40.8%. Finally, a prototype is developed and platform tests are performed to validate the theoretical analysis results.

## 1. Introduction

With the increasing growth of global energy demand and increased awareness of environmental protection, wave power production technology has emerged as a renewable energy research hotspot due to its abundant of resources, environmental friendliness, and consistent reliability. Research and optimization of wave energy converters (WECs) are critical for supporting renewable energy development [1].

Wave energy conversion systems are primarily categorized into three types: oscillating water columns, overtopping wave energy convertor, and oscillating buoy wave energy convertor [2]. The oscillating buoy wave energy convertor is a piece of equipment that harvests wave energy by utilizing the heaving motion of the oscillating float caused by wave forces [3]. As discussed in [4] and [5], typical WECs have drawbacks such as low force density, huge machine size, and costly maintenance requirements. Traditional mechanical WECs achieve speed increase by integrating gearboxes, resulting in extra energy losses and high maintenance costs [6]. Another type of direct-drive point absorber wave energy producing system uses a linear generator, which simplifies the design [7]. However, when power output increases, the weight and volume of the linear generator increase. Halbach magnetized magnetic screw (HMMS), axially-magnetized magnetic screw (AMMS), and radially-magnetized magnetic screw (RMMS) are the three possible magnet configurations for the magnetic screw (MS) [8].

**Fig 1:**
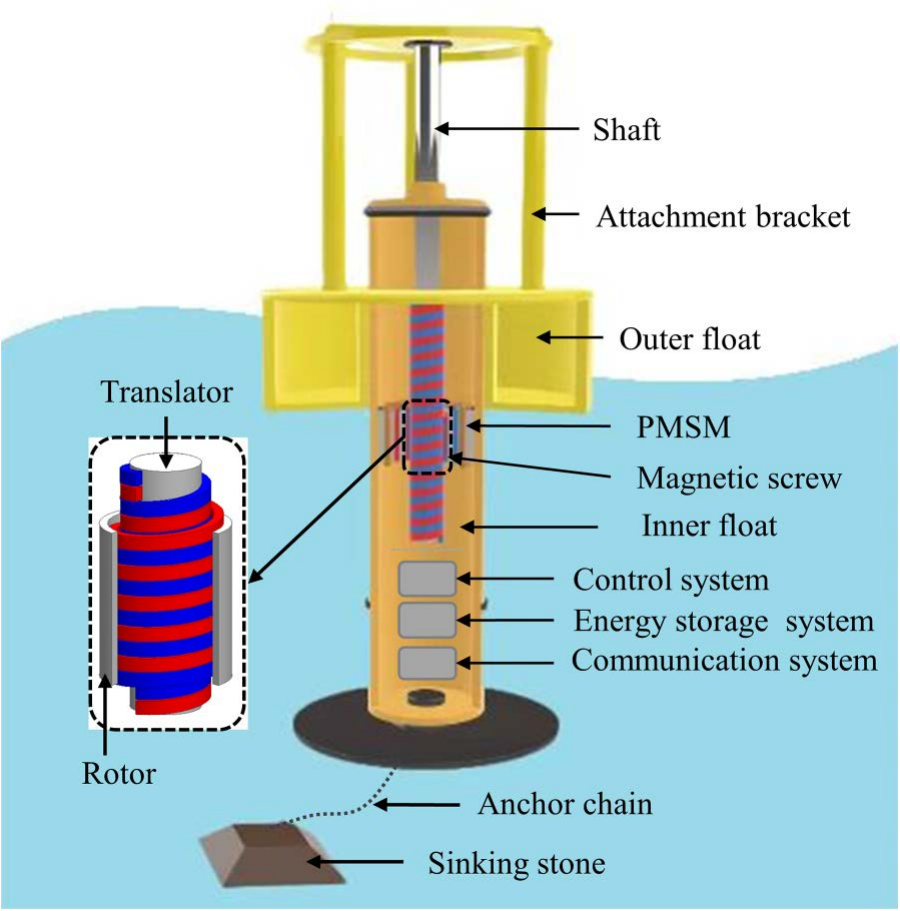
WECs with MS replacing mechanical gearbox.

To address these challenges and enhance transmission reliability, the MS is employed instead of the mechanical screw used for WECs [9], the model is shown as Fig 1. The composite design of the rotor and the permanent magnet synchronous motor (PMSM) rotor not only simplifies the structure of the energy conversion system but also reduces the overall size of WECs. Moreover, the embedded HMMS structure effectively shields permanent magnets (PMs) material from seawater corrosion significantly extending the service life of the equipment. The MS, a type of magnetic gear is based primarily on the interaction of PMs to convert torque and speed without physical touch. The MS offers high transmission efficiency, compactness, and low maintenance [10]. Introduced a new structure of MS specifically designed for WEC applications. This design seeks to convert low-speed linear wave motion into high-speed rotating motion, making it an essential component in WECs. In [11], a unique radially magnetized MS construction is proposed, utilizing discrete PM arcs to approximate the ideal helical pole, thereby simplifying the manufacturing and assembly processes while preserving performance close to that of the ideal helical pole. In [7], an embedded magnet topology is proposed for the first time, which avoids some disadvantages of traditional surface-mounted magnets in the assembly process, and improves the structural stability and assembly convenience of the MS. In [12], a novel variant of the segmented modulated magnetic flux screw is introduced. This approach mitigates the complexity and expense of PM processing, streamlines the system architecture, and diminishes the overall dimensions of the equipment. In [13], a new type of magnetic field modulation screw is proposed, which achieves a high torque ratio by selecting a reasonable number of poles. The contribution of air gap magnetic density harmonics to torque and thrust is analyzed by using three-dimensional Maxwell tensor method. Overall, research on MS technologies for WECs is advancing, with an emphasis on improving efficiency, performance, and sustainability.

Furthermore, recent research on MS has predominantly focused on topological variants and control strategies, with relatively limited attention to multi-objective optimization [14–16] linearized an HMMS–WEC model and derived its transfer function but addressed only a single objective, thereby neglecting the trade-offs among competing performance metrics [17] experimentally validated a 1 kW discrete-Halbach MS prototype; however, the design process relied heavily on manual parameter tuning. Conventional frameworks typically collapse multiple objectives into a single weighted sum, necessitating repeated runs with varying weighting factors to approximate a Pareto front [18]. While surrogate-assisted evolutionary algorithms mitigate this limitation, their convergence remains slow when a full finite-element analysis (FEA) is required at each iteration. To accelerate design exploration, analytical field models for Halbach screws have been extended to arbitrary pole-pair combinations, offering accurate initial estimates for subsequent FEA refinement [19]. A few studies have also explored surrogate-based optimization for magnetic gears: [20] applied a co-Kriging multi-fidelity model to a dual-modulator coaxial magnetic gear design, and [21] utilized response surface (RS) models for a vernier machine—demonstrating the potential for cross-application of these techniques. Many prior designs either optimized a single objective or converted multiple objectives into a scalar value through weighting, potentially overlooking critical trade-off solutions. The interplay between maximizing force density and achieving smooth sinusoidal magnetic flux remains underexplored. As a result, current HMMS designs necessitate a dedicated multi-objective optimization framework capable of addressing the nonlinear magnetic interactions and practical constraints inherent in HMMSs for WECs.

In the INFO-KELM model, the KELM serves as a fast and stable learning core, while the INFO algorithm, proposed in 2022 [22], optimizes the regularization coefficient and kernel parameters of the KELM. Given that the KELM output is highly sensitive to these hyperparameters, the INFO algorithm dynamically adjusts search parameters and updates candidate solutions via weighted averages, adaptively balancing exploration and exploitation during optimization. This accelerates convergence to the optimum. As a result, the INFO-KELM model achieves higher predictive accuracy and stability, identifying superior design solutions with fewer evaluations compared to standard evolutionary algorithms.

The remainder of the paper is structured as follows. Section 2 introduces the structure and operation principle of HMMS. In Section 3, the INFO-KELM model is established and the essential parameters of the model are optimized based on the findings of the thorough sensitivity analysis. The final optimization result is obtained. In Section 4, the electromagnetic performance of the model is evaluated using finite element analysis. Section 5 describes and evaluates the prototype and test platform. Section 6 concludes the article.

## 2. Topology of the HMMS

**Fig 2:**
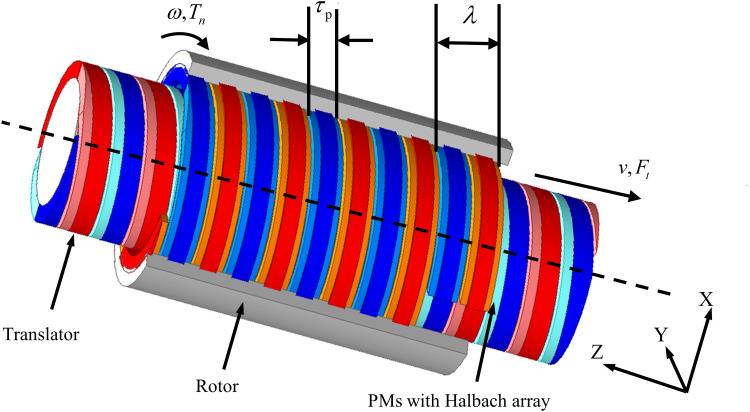
Structure of proposed HMMS.

The topology of the proposed HMMS is presented in Fig 2. It is observed that the main structure of HMMS consists of a translator and a rotor. Both core components are equipped with discrete, segmented PMs arranged in a spiral configuration to mimic the shape of spiral PMs. The proposed HMMS features an improved Halbach structure for both the rotor and translator, utilizing helical Halbach magnet arrays with PMs. This Halbach structure exhibits a magnetic focusing effect. The air gap spacing of the translator comprises radial and axially magnetized PMs, while the rotor poles consist of three single-layer heterogeneous Halbach magnet arrays. When there are two poles per pole in the Halbach magnet array, the increase in air gap magnetic density is not immediately apparent. Nonetheless, the sinusoidal magnetic density escalates with the augmentation of individual magnets. As the quantity of magnets increases, the intricacy of machining and cutting magnetic steel also escalates. Therefore, considering manufacturing constraints, two equally thick magnets are selected on the side with fewer poles in the translator, while three unequally thick magnets are chosen on the side with fewer poles in the rotor. Each pole is thus composed of three single magnets with different magnetic orientations. When the translator moves smoothly along a straight line in response to wave motion, the rotor exhibits distinct dynamic characteristics.

**Fig 3:**
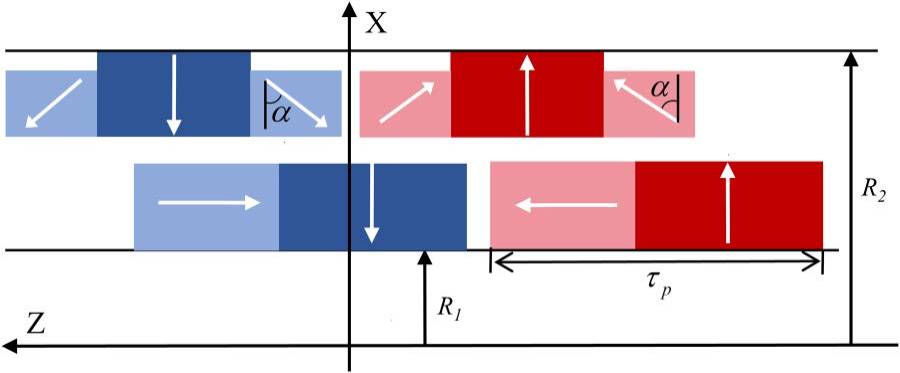
Proposed HMMS topology showing exploded view of translator and rotor with Halbach arrays.

The optimization of the magnetic field is achieved through an improved Halbach magnetization configuration, which is one of the key features of the structure. The axial thrust produced by HMMS is increased by this configuration, which also concentrates the magnetic field in a particular direction and increases the magnetic flux density in the air gap. The PM magnetization mode, as shown in Fig 3, is configured into three distinct sections, each with varying widths and thicknesses. In this design, *R*_*1*_ represents the outer diameter of the translator, while *R*_*2*_ denotes the inner diameter of the rotor. Each pole on the translator is divided into two PM segments: one radially magnetized and the other axially magnetized. Three Halbach arrays with varying widths and thicknesses are separated into each pole of the rotor’s PM. In particular, tiny blocks on the left and right possess a magnetization angle α, while the central PM segment exhibits radial magnetism. A magnetization array with *α *= 45° is adopted to achieve magnetic focusing via the Halbach magnetization structure.

In the proposed HMMS, the magnetic field topology is determined by the spatial orientation of PMs arranged in a helical configuration. This arrangement generates a unidirectional magnetic flux distribution, which is critical for efficient linear thrust force generation in wave energy conversion systems.

For Halbach magnetized PMs, each pole is divided into multiple sections, and the magnetization direction of each section is determined by the angle α. The remanent magnetization vector components in cylindrical coordinates for the Halbach array are expressed as follows [23]:


$$M_{R}=\sum_{n=1,3,5}^{\infty }M_{Rn}\left(n\right)\cos\left[np\left(\alpha -\alpha_{0}\right)\right]$$ (1)

$$M_{A}=\sum_{n=1,3,5}^{\infty }M_{An}\left(n\right)\sin\left[np\left(\alpha -\alpha_{0}\right)\right]$$ (2)

Using the Fourier series expansion approach, these two groups of equations describe the variation laws of the radial component *M*_*rn*_ and the tangential component *M*_*θn*_ of the magnetization vector *M* of the PMs in HMMS with respect to spatial position. *M*_*rn*_ denotes the distribution of the magnetization vector in the radial direction and directly influences the radial intensity of the air-gap magnetic field. *M*_*θn*_ represents the distribution of the magnetization vector in the circumferential direction and determines the tangential coupling capability of the magnetic field.


$$M_{Rn}=\frac{4Br}{n\mu_{0}\pi }\sin\left(\frac{n\pi }{2a}\right)\cdot \left\{1+\sum_{i=2}^{m}\cos\left[\frac{\left(i-1\right)n\pi }{a}\right]\cos\left[\frac{\pm (i-1)\pi }{a}\right]\right\}$$ (3)

$$M_{An}=\frac{4Br}{n\mu_{0}\pi }\sin\left(\frac{n\pi }{2a}\right)\cdot \left\{\sum_{i=2}^{m}\sin\left[\frac{\left(i-1\right)n\pi }{a}\right]\sin\left[\frac{\pm (i-1)\pi }{a}\right]\right\}$$ (4)

where *p* is the amount of pole pairs, *B*_*r*_ is the remanence of PMs, *a i*s the amount of PM segments per pole, *i* is the *i*th piece of PM segments per pole and *α* is the magnetization angle between each PMs and the *x*-axis. (1)-(4) lead the optimization of segmentation and magnetization orientation of PMs to augment target harmonics and mitigate distorted harmonics.

The components of the magnetic field can be described as circumferential and axial function expressions with constant radius and position. In summary, the HMMS successfully realizes the transfer process of mechanical energy without changing the energy form. In particular, the magnetic field within the air gap serves as an intermediate for mechanical energy transmission between the actuator and the rotor. The interaction of the magnetic fields generated by the rotor and actuator creates this magnetic field. According to the principle of energy conservation, in idealized conditions, if we ignore all kinds of losses that may occur along the energy transmission path, then the entire energy transmission process will strictly follow the conservation law.


$$G=F_{t}/T_{n}=\omega /v=2\pi /\lambda$$ (5)

where *λ* is the screw pitch of the PM. The transmission ratio depends on fixed geometric parameters. (5) delineates the mathematical correlation between the pitch λ of the HMMS and energy transfer, illustrating the conserved transfer of mechanical energy under optimal conditions. The existing high-performance MS system ensures stable energy transmission due to its constant transmission ratio. Considering the requirement for the ratio between wave speed and rotating motor speed, if the linear speed generated by the wave is 0.33 m/s and the rotating motor speed is 1000 rpm, the pitch of the HMMS is determined to be 10 mm in this design based on the transmission ratio calculation formula.

## 3. Multi-objective optimization

**Fig 4:**
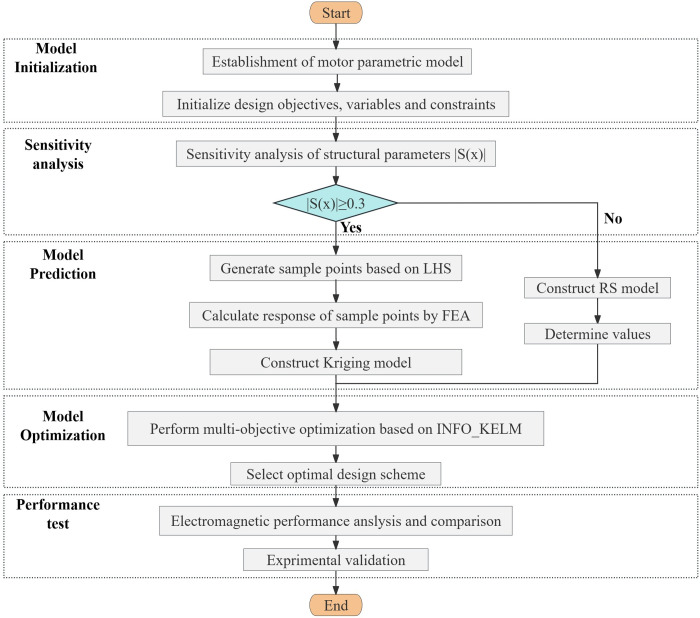
Multi-objective optimization design process of proposed HMMS.

Due to the complex structure of PMs, more parameters need to be considered in the optimization of the proposed HMMS, which increases the difficulty of optimization. To ensure optimal performance of the HMMS across a range of operating conditions, it is essential to consider multiple optimization criteria. The three basic design objectives are the maximizing of thrust force, the augmentation of air gap magnetic flux density, and the reduction of sinusoidal distortion in the air gap magnetic flux density. In this study, to avoid obvious local saturation and make full use of soft magnetic materials, the maximal magnetic flux density of HMMS is limited to 2T or less. The multi-objective intelligent optimization algorithm can take into account the requirements of multiple objectives, it can effectively approach the global optimal solution or the satisfactory optimization solution close to the global optimal. The proposed multi-objective optimization model is shown in Fig 4.

1) Model initialization stage: For the target object to be studied in this paper, design variables are determined and parameterized models are established. Determine optimization objectives, optimization variables and constraints according to engineering requirements or expected improvement direction. Due to the high-dimensional design space of the model to be optimized, a thorough sensitivity analysis is implemented to decrease computational effort by categorizing the design variables into sensitive and non-sensitive dimensions.
2) Model prediction stage: Kriging agent model and RS model are established for sensitive parameters and non-sensitive parameters of HMMS respectively to achieve accurate and rapid prediction of all optimization objectives. These surrogate models capture the relationships between design parameters and objectives and remain fixed once established; they serve as static predictive models during the optimization. A combination of the agent model and the finite element model is used to achieve the following goals: to shorten the optimization design cycle, to reduce calculation time and cost, to strike a balance between calculation time and calculation accuracy, and to shorten the calculation time.
3) Model optimization stage: in the key stage of model optimization, this paper proposes a HMMS parameter optimization model based on INFO-KELM. Since regularization coefficient and kernel function parameters have a crucial impact on the performance of KELM, this paper uses INFO algorithm to optimize the selection of KELM parameters, and thus builds an efficient INFO-KELM parameter optimization model.

The RS model is utilized to manage non-sensitive parameters in HMMS. Although these parameters have a relatively minor impact on the optimization objective, their potential influence on overall performance must still be considered during the optimization process. By establishing an approximate relationship between the parameters and the optimization objective, the RS model efficiently predicts the effects of non-sensitive parameters, thereby reducing computational costs. Meanwhile, the INFO-KELM model focuses on optimizing sensitive parameters in HMMS, which significantly influence the optimization objective. The INFO algorithm enhances the prediction accuracy of the INFO-KELM model for sensitive parameters by fine-tuning the kernel function parameters and regularization coefficients, thus improving the overall performance of HMMS. Through collaborative operation, the RS model and the INFO-KELM model ensure that both sensitive and non-sensitive parameters are comprehensively accounted for during the optimization process. This approach enables the optimization process to dynamically adjust parameters, ensuring the achievement of the optimization objective. Such a dynamic adjustment mechanism not only improves optimization efficiency but also ensures the accuracy and reliability of the optimization results.

### 3.1. Parameter classification and sensitivity analysis

**Fig 5:**
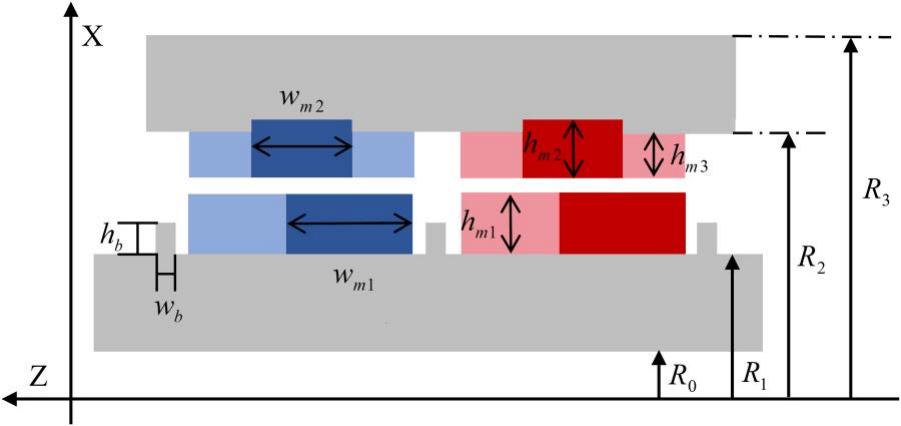
Design parameters of proposed HMMS.

As shown in Fig 5, the constant parameters related to the machine geometry are shown in detail. In order to accurately define and identify the geometric features of the proposed machine, it is necessary to conduct a thorough and detailed evaluation of the 15 key design variables.

To precisely define the geometry of the proposed HMMS, a comprehensive evaluation of 15 core design variables is required. On this basis, reasonable constraints and optimization of design parameters are carried out, as shown in Table 1 for specific details. These geometric parameters can be further subdivided into different categories according to their properties and functions. The primary category is the original design parameters, which form the basic framework of the HMM geometry. The HMMS created in this work must transform wave linear motion to generator rotational motion based on the transmission ratio calculations in the operating principle. The pole pitch is set to 10 mm, and assuming a wave speed of 0.4m/s, the HMMS rotor rotates at 1200 rpm. The outer radius of the rotor is set at 35 mm to accommodate the volume limit of WEC devices. The thickness of the translator backing plate and the rotor backing plate are closely connected characteristics that influence total structural strength. Because of the HMMS’s mechanical strength limitations, these two parameters cannot be too small. As a result, the minimum size required to achieve the strength specifications is calculated throughout the design phase. In this work, they are measured as 5 mm and 3 mm, respectively. The PM thickness is closely connected to its demagnetization performance. A thin PM will demagnetize the HMMS, lowering its reliability. As a result, the minimum PM thickness is set at 3 mm.

**Table 1:** Design variables.

Parameters	Definitions	Units	Range/Value
*R*_*0*_	Inner radius of translator	mm	5
*R*_*1*_	Outer radius of translator	mm	(16, 20)
*R*_*2*_	Inner radius of rotor	mm	(28, 34)
*R*_*3*_	Outer radius of rotor	mm	(36, 42)
*w*_*m1*_	Width of radial translator PMs	mm	(2,8)
*h*_*m1*_	Thickness of translator PMs	mm	(3,6)
*w*_*m2*_	Width of high rotor PMs	mm	(3,6)
*h*_*m2*_	Thickness of high rotor PMs	mm	(3,6)
*h*_*m3*_	Thickness of low rotor PMs	mm	(3,6)
*w*_*b*_	Width of bulge	mm	(0,1)
*h*_*b*_	Thickness of bulge	mm	(0,1)
*l*_*a*_	Length of airgap	mm	2
*p*	Number of poles		5
*τ*_*p*_	Length of pole	mm	9
*λ*	Pole pitch	mm	10

The aim of sensitivity analysis is to assess the impact of structural attributes on the motor performance of HMMS, and the size of sensitivity indicators can be used to group structural parameters, which can then be processed in various ways to ensure that the optimal result is obtained while significantly reducing calculation time and cost.

Based on comprehensive sensitivity analysis, detailed sensitivity analysis results are shown in Table 2. The sensitivity of each parameter is classified into two categories: strong sensitivity and weak sensitivity, to accurately evaluate the sensitivity characteristics of the model’s structural parameters.

**Table 2:** Different hierarchical structure parameter levels.

Level	Structural parameters
Strong-sensitive	$w_{m1}$,$w_{m2}$,$h_{m1}$,$h_{m2}$
Weak-sensitive	$R_{1}$,$R_{2}$,$h_{m3}$,$w_{b}$,$h_{b}$


$$S_{com}\left(x_{i}\right)=\lambda_{1}\left|S_{F_{\max}}\left(x_{i}\right)\right|+\lambda_{2}\left|S_{Bz}\left(x_{i}\right)\right|+\lambda_{3}\left|S_{kB}\left(x_{i}\right)\right|$$ (6)

where *S*_*com*_*(x*_*i*_) represents the comprehensive sensitivity index of the *i*th design variable. This index systematically evaluates the extent to which design variables influence various design objectives. Among them, *λ*_*1*_, *λ*_*2*_ and *λ*_*3*_ are weight coefficients assigned to three different design objectives, and these coefficients quantitatively reflect the relative importance of each objective on the performance of the HMMS. Based on (a) theoretical priority: thrust maximization is the primary requirement for energy capture in WECs, as it directly determines WECs efficiency, and (b) empirical regression: pre-optimized FEA data indicate that thrust contributes approximately 62% to the overall efficiency gain. To ensure objectivity, this study adopts the entropy weight method to calculate the initial weights based on the dispersion of FEA results, followed by normalization. The final values of *λ*_*1*_, *λ*_*2*_ and *λ*_*3*_ are determined as (0.6, 0.3, 0.1). The sensitivity design variable to three design objectives is denoted by *S*_*Fmax*_*(x*_*i*_), *S*_*Bz*_*(x*_*i*_), and *S*_*kB*_*(x*_*i*_), respectively. It is possible to ascertain the entire sensitivity index of every design variable by Eq 6. The sensitivity analysis results of HMMS are illustrated in Fig 6.

**Fig 6:**
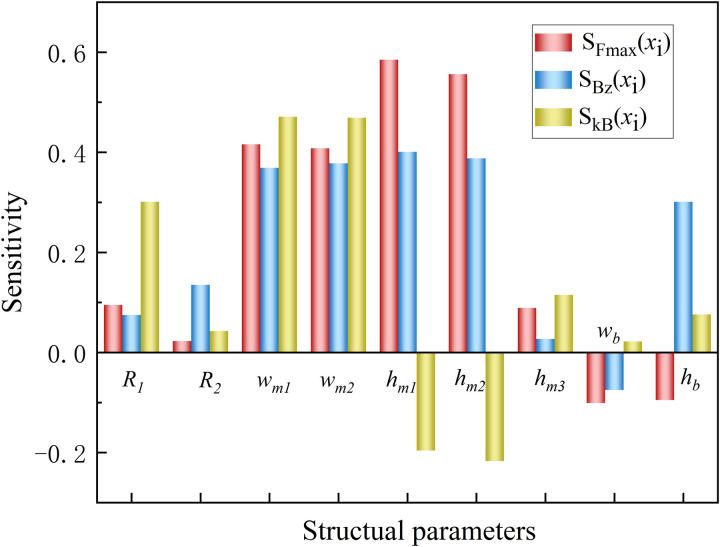
Sensitivity analysis results of HMMS.

The optimization process can be rendered more effective and efficient by assigning structural parameters to different optimization levels in accordance with their sensitivity fluctuations. According to the results of the sensitivity analysis, design variables can be categorized into two primary tiers.

The first level includes those design variables with high sensitivity, that is, when the comprehensive sensitivity index *S*_*com*_*(x*_*i*_) is greater than 0.3, the design variable is considered to have a significant impact on system performance. Specifically, *w*_*m1*_, *w*_*m2*_, *h*_*m1*_ and *h*_*m2*_ are classified as strong-sensitive design variables that require special attention and adjustment during design optimization. The second level includes those design variables with relatively low sensitivity, that is, when the comprehensive sensitivity index *S*_*com*_*(x*_*i*_) is less than 0.3, it is considered that the design variable has a weak impact on the system performance. Under this category, *R*_*1*_, *R*_*2*_, *h*_*m3*_, *w*_*b*_ and *h*_*b*_ are considered weak-sensitive design variables that may not require frequent adjustments during optimization or can be considered as minor factors. The results of different hierarchical structure parameter levels is shown in Table 2.

### 3.2. Kriging and RS Model

The Kriging model is an unbiased estimation model designed to minimize estimate variance. Due to the unknown complexity of the prediction model, it is infeasible to generate an appropriate surrogate model from a single data point; therefore, the model must be continuously updated to meet evolving requirements [24].

LHS is a sort of stratified sampling derived from Latin Square Arrays. A Latin square array of size *n × n* with n elements requires each element to appear in a different row and column. The LHS is a higher-dimensional expansion of the Latin square array. Because the optimization results are unknown prior to optimization, there is no obvious preference for the range of optimization variables, hence the sampling method assigns each optimization variable to a uniform distribution in its parameter space. A Latin hypercube sampling with m variables and n samples divides each variable into *n* equal probability subregions, from which a random value is chosen. Finally, the m dimensions are combined to create a sample point. The LHS matrix is created using the following formula


$$\underset{D}{\min}{\left\{\sum_{i=1}^{n-1}\sum_{j=i+1}^{n}\frac{1}{d^{k}\left(x_{i},x_{j}\right)}\right\}}^{1/k}$$ (7)

$$d\left(u,v\right)={\left(\sum_{i=1}^{n}{\left|u_{i}-v_{i}\right|}^{S}\right)}^{1/S}$$ (8)

**Fig 7:**
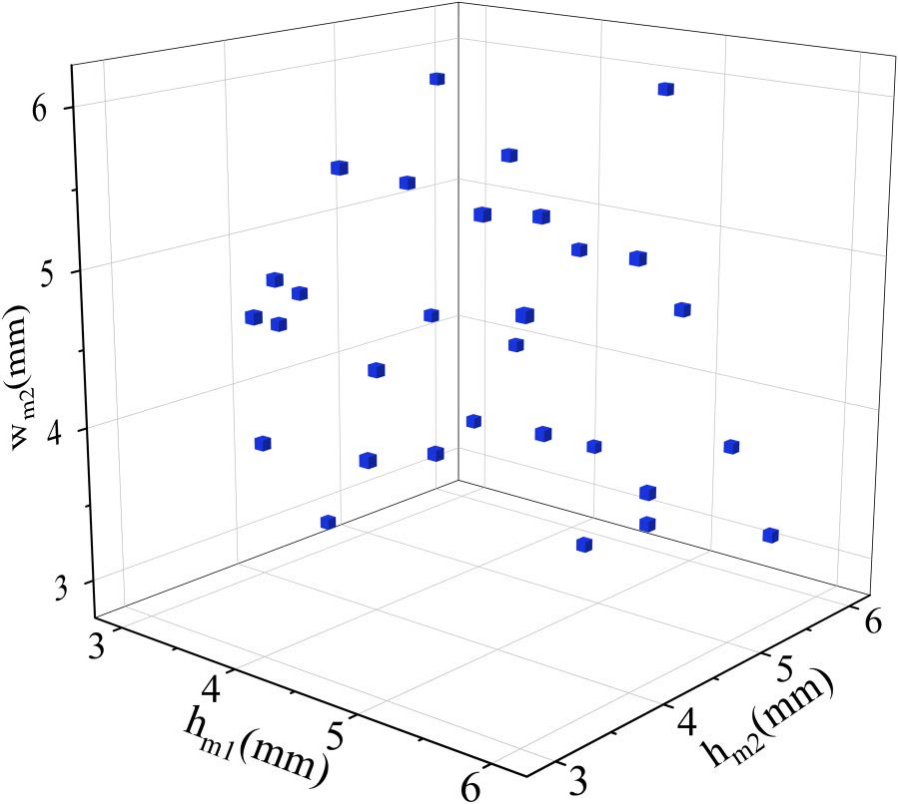
LHS distribution of three parameters.

In order to match the data features with the fewest number of samples and maximize the distribution of sample points in the sample space, this paper uses an improved multi-criteria LHS method that takes into account the uniformity and orthogonality of spatial distribution to improve surrogate model accuracy while also reducing sample size. The enhanced LHS considers the minimum distance and correlation coefficient between sample points to assess spatial distribution uniformity and orthogonality. To aid optimization design, a composite indicator is employed to depict the spatial distribution. The distribution points of four strong-sensitive parameters are generated using the LHS approach, the number of Latin hypercube layers is set to 30, the number of parameters is set to 4, and the corresponding ranges of parameters are 2 < *w*_*m1*_ < 8, 3 < *w*_*m2*_ < 6, 3 <* h*_*m1*_ < 6 and 3 < *h*_*m2*_ < 6. A set of homogenized initial values of variables are created. Fig 7 displays the generated variable value distribution using *w*_*m2*_, *h*_*m1*_ and *h*_*m2*_ as examples.

Kriging model regards prediction *y*_*x*_ as a random function, including regression part and random part, and its mathematical model is expressed as


$$y_{x}=\mu^{T}\left(x\right)\beta +z\left(x\right)$$ (9)

where *μ*^*T*^*(x)* is the parametric polynomial model, *μ(x)=[μ*_*1*_*(x),…, μ*_*n*_*(x)]*^*T*^ is a polynomial basis function vector used to provide a global approximation of Kriging model, *n* is the number of polynomials. *β=[β*_*1*_*,…, β*_*n*_*]*^*T*^ is the polynomial regression coefficient corresponding to *μ(x), μ*^*T*^*(x)β* is in the form of ordinary, so *μ*^*T*^*(x)β=β*_*0*_, *x(x)* is a Gaussian random process with a mean of 0, and its covariance can be expressed as


$$Cov\left[z\left(x^{i}\right),z\left(x^{j}\right)\right]=\sigma^{2}R\left(x^{i},x^{j},\theta \right)$$ (10)

where σ is the standard deviation of the random error, *R(x*^*i*^*, x*^*j*^*, θ*) *i*s the spatial correlation function expressed as any two sample points, when the Gaussian function is adopted, its expression is


$$R_{\theta }\left(x^{i},x^{j},\theta \right)=\exp\left(-\sum_{a=1}^{n}\theta_{a}{\left(x_{a}^{i}-x_{a}^{j}\right)}^{2}\right)=\prod_{n=a}^{n}\exp\left(-\theta_{a}{\left(x_{a}^{i}-x_{a}^{j}\right)}^{2}\right)$$ (11)

**Fig 8:**
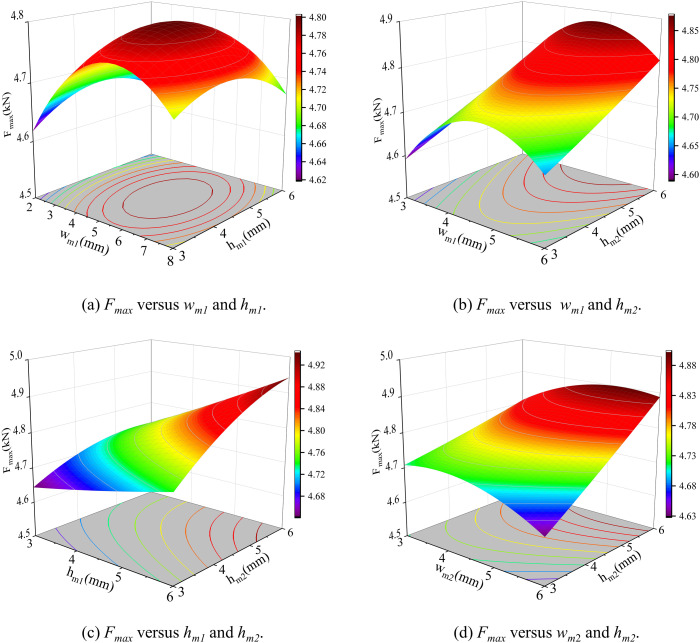
Response of the objective function *F*_*max*_ to different key parameters.

The input parameters of the sample include *w*_*m1*_, *w*_*m2*_, *h*_*m1*_, and *h*_*m2*_, while the output parameters are three target parameters derived using the finite element analysis (FEA). The input parameters encompass several key variables, whereas the output parameters consist of the three core target parameters precisely obtained through FEA. To construct the prediction model, the Kriging model is adopted, with 30 sample points of Latin Hypercube sampling (LHS) used to improve efficiency. Taking the objective function – maximum thrust as an example, the relationship of strong-sensitive parameters is presented, as depicted in Fig 8. Kriging model shows a strong forecasting ability, which can not only accurately capture and predict the global trend between the input parameters and the target parameters, but also accurately describe the local feature change law. This result shows that the model can effectively reveal and quantify the complex effects of input parameters on target performance parameters even with a limited number of samples.

The secondary RS model established for the five weak-sensitive structural parameters can be written as


$$\overset{\wedge }{y}\left(x\right)=\beta_{0}+\sum_{i=1}^{k}\beta_{i}x_{i}+\sum_{i=1}^{k}\beta_{ii}{x_{i}}^{2}+\sum_{i<j}\beta_{ij}x_{i}x_{j}$$ (12)

where *x*_*i*_ and *x*_*j*_ are the design variables, and the *β*_*0*_*, β*_*i*_*, β*_*ii*_*, β*_*ij*_ are regression coefficients obtained by ordinary least squares on a LHS design. This quadratic form captures linear, interaction, and curvature effects while remaining inexpensive to evaluate.

Sensitivity analysis revealed that the five weakly-sensitive variables exhibit smooth, approximately quadratic influence on the objectives, whereas the four strongly-sensitive variables demonstrate significant non-linearity and coupling effects. Consequently, the RS model is employed for the weak-sensitive parameters because a quadratic surface is sufficiently accurate, requires fewer sample points, and reduces computational cost by orders of magnitude.

**Fig 9:**
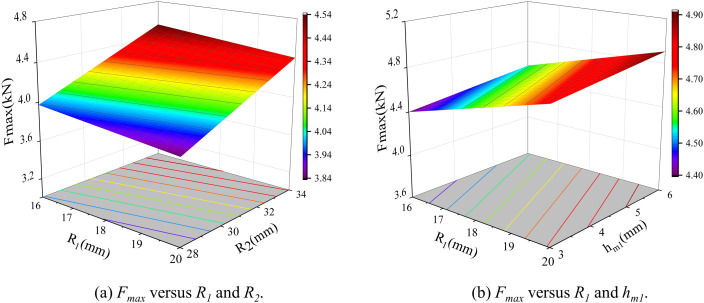
Response of the objective function *F*_*max*_ to different key parameters.

The significant interaction terms are observed only in the most relevant pairs of variables, suggesting an absence of strong nonlinear coupling among the weakly sensitive parameters. This is also why the RS model can maintain a high level of accuracy. Given that the RS model is based on a global quadratic form, it may exhibit reduced accuracy when weakly sensitive variables exceed the training range or local higher-order effects emerge. The accuracy requirements have been validated through error statistics within the constraint interval in this study. Fig 9. illustrates the responses of two sets of maximum thrust under varying parameters. Through analysis of the RS model, the values of the five weak-sensitive parameters were established.

### 3.3. Multi-objective optimization based on INFO-KELM

The HMMS optimization design problem is nonlinear, and its change trend prediction is difficult, which complicates analysis. Neural networks perform well in nonlinear issues. However, limitations in actual applications include limited training speed and insufficient parameter flexibility. Additionally, a KELM is employed to model and improve the previously defined condition. The INFO approach is employed to optimize the regularization coefficient and kernel function parameters, hence improving the accuracy of the classification recognition function of the KELM [25].

#### 3.3.1. Kernel extreme learning machine (KELM).

KELM is a method that integrates extreme learning machines with kernel learning algorithms. When creating a learning standard in machine learning, the sample points must be multiplied one by one, and the sample points in the vector space can be generated using kernel functions. Feature mapping transforms the sample from a low-dimensional space to a high-dimensional feature space, allowing it to be separated linearly.

**Fig 10:**
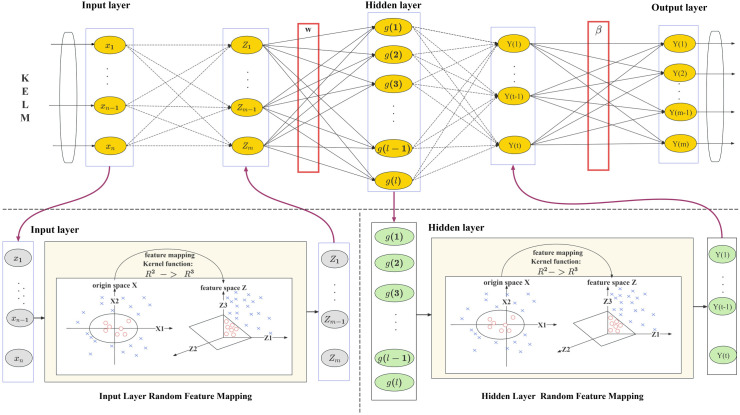
KELM model training diagram within the INFO-KELM optimization process.

Fig 10 illustrates the training procedure of the KELM model, along with the feature mapping processes in the input and hidden layers. The KELM model addresses nonlinear problems by transforming input data into a high-dimensional feature space and performing linear learning within this space through the use of kernel functions. This approach not only enhances the efficiency of the model training process but also significantly improves its generalization capability.

The input layer acquires the original data *x*_*1*_, *x*_*2*_,..., *x*_*n*_, and transforms it into a high-dimensional feature space *Z*_*1*_, *Z*_*2*_,..., *Z*_*m*_ via the stochastic feature mapping. This approach excludes weight training, hence considerably diminishing the time complexity of model training. Each node in the hidden layer processes the features transformed by the input layer via the kernel function K(·), producing activation values *a(1)*, *a(2)*,..., *a(l)*. The activation values are subsequently utilized for the linear combination of the output layer. The output layer linearly integrates the activation values from the hidden layers using the weight β to get the final anticipated outputs *Y(1)*, *Y(2)*,..., *Y(m)*. The illustration additionally depicts the stochastic feature mapping procedure of the input layer and the hidden layer. The random feature mapping of the input layer transforms the original space into the feature space *Z*, while the random feature mapping of the hidden layer further converts the feature space *Z* into another feature space *Z’* to enable linear learning within this space.

ELM employs random feature mapping and least-squares optimization, where the least squares method is utilized to learn from the mapped data. Then the least square method is used to learn the mapped data. Compared with traditional neural networks, ELM does not need to perform iterative parameter adjustment, but can complete the training through a single forward propagation, so it has higher efficiency and scalability. The ELM model of random mapping can be expressed as


$$T=\sum_{i=1}^{l}\beta g\left(w_{i},b_{i},x\right)$$ (13)

where *T* is the output matrix of the ELM target data, *g(·)* is the activation function of the hidden layer neurons, *w*_*i*_ is the input layer weight matrix, *b*_*i*_ is the hidden layer bias matrix, *β* is the output layer weight matrix. By training the model parameters, ELM makes the model output infinitely approximate the training sample objective function such as the formula


$$Min{\left\|H\beta -T\right\|}^{2},\beta \in R^{l\times m}$$ (14)

where *H* is the output matrix of the hidden layer, *T* is the target matrix of the training data. The output matrix of the ELM model with *l* hidden layer nodes can be expressed as


$$H={\left[h\left(x_{1}\right),\ldots ,h\left(x_{n}\right)\right]}^{T}=\left[\begin{array}{ccc} {}*20ch_{1}\left(x_{1}\right) & \cdots & h_{L}\left(x_{1}\right)\\ \vdots & \cdots & \vdots \\ h_{1}\left(x_{n}\right) & \cdots & h_{l}\left(x_{n}\right) \end{array}\right],T={\left[t_{1},\ldots ,tn\right]}^{T}$$ (15)

The input weight and bias of the ELM are allocated randomly, resulting in instability in its predictive performance and imposing constraints on prediction accuracy. In order to overcome this shortcoming, kernel function is introduced to optimize the traditional ELM algorithm. The KELM, derived from the original extreme learning machine, employs kernel mapping instead of random mapping to transform low-dimensional input into high-dimensional space for inner product operations. Compared to the original extreme learning machine, this substantially reduces computational complexity and enhances output stability and generalisability. The kernel matrix of KELM is defined as follows when the concealed layer mapping is unknown after the kernel function is introduced into ELM.


$$\left\{ \begin{array}{c} \Omega_{KELM}=HH^{T}\\ \Omega_{KELMi,j}=h\left(x_{i}\right)\cdot h\left(x_{j}\right)=K\left(x_{i},x_{j}\right) \end{array}\right.$$ (16)

where *Ω* represents the kernel matrix, *K(·)* represents the kernel function, *i* and *j* are random values. Use *ℎ(*x)H^*T*^ replaces the expression with the kernel *K*(*x*𝑖, *x*𝑗)


$$h\left(x\right)H^{T}={\left(\begin{array}{c} K\left(x,x_{1}\right)\\ \vdots \\ K\left(x,x_{n}\right) \end{array}\right)}^{T}$$ (17)

Using the regularization coefficient C in the optimization stage, the output of the KELM can be expressed as


$$f_{KELM}\left(x\right)={\left(\begin{array}{c} K\left(x,x_{1}\right)\\ \vdots \\ K\left(x,x_{n}\right) \end{array}\right)}^{T}{\left(\frac{1}{C}I+\Omega_{KELM}\right)}^{-1}T$$ (18)

Kernel parameters and regularization coefficients are critical factors influencing the complexity and stability of HMMS parameter samples when mapped to high-dimensional feature space. The INFO method is employed to optimize the parameters of the KELM. This method is employed to address the issue of inadequate model accuracy caused by arbitrary parameter selection. This optimization enhances the precision of HMMS parameter estimation.

#### 3.3.2. Weighted mean of vectors algorithm (INFO).

The INFO algorithm aims to find the optimal or near-optimal answer in the large search space by iteratively updating the positions of vectors in the population using the weighted average approach. By dynamically adjusting parameters and calculating weighted average values, the algorithm can adapt to a variety of complex optimization problems, especially for constrained optimization problems.

The INFO algorithm starts with the random initialization of a population including many vectors that represent potential solutions inside the issue search space. The method design incorporates two essential adaptive parameters: the weighted average factor (*δ*) and the scaling factor (*σ*). The two parameters are dynamically adjusted in the iterative process of the algorithm without manual intervention by users, thus realizing the adaptive optimization of parameters. This feature enables INFO algorithm to efficiently balance exploration and utilization in complex search space to find optimal or near optimal solutions.

(1) Updating rule stage

New vectors are created by utilizing mean-based criteria in conjunction with a convergence acceleration approach. This is executed to enhance the diversity of the population. The process starts by randomly choosing a subset of vectors from the population, computing their weighted average, and subsequently updating the position of the current vector based on this calculated average. The formula for the mean rule is given as follows:


$$MeanRule=r_{1}\times WM1_{l}^{g}+\left(1-r_{1}\right)\times WM2_{l}^{g},l=1,2,\ldots ,n$$ (19)

$$WM1_{l}^{g}=\delta \times \frac{w_{1}\left(x_{a1}-x_{a2}\right)+w_{2}\left(x_{a1}-x_{a3}\right)+w_{3}\left(x_{a2}-x_{a3}\right)}{w_{1}+w_{2}+w_{3}+\varepsilon }+\varepsilon \times rand,l=1,2,...,n$$ (20)

where *l* is a random number between [1, n], *g* is the amount of iterations; *r*_*1*_ is a random number between [0, 0.5], ε is a constant with a very small value, *a*_*1*_, *a*_*2*_, *a*_*3*_ are the randomly chosen distinct integers between (1, N], *rand* is a random value with a normal distribution, *w*_*1*_, *w*_*2*_, *w*_*3*_ are weighting functions. The weighting functions are as follows.


$$\left\{ \begin{array}{c} w_{1}=\cos\left(\left(f\left(x_{a1}\right)-f\left(x_{a2}\right)\right)+\pi \right)\times \exp\left(-\left|\frac{f\left(x_{a1}\right)-f\left(x_{a2}\right)}{\omega }\right|\right)\\ w_{2}=\cos\left(\left(f\left(x_{a1}\right)-f\left(x_{a3}\right)\right)+\pi \right)\times \exp\left(-\left|\frac{f\left(x_{a1}\right)-f\left(x_{a3}\right)}{\omega }\right|\right)\\ w_{3}=\cos\left(\left(f\left(x_{a2}\right)-f\left(x_{a3}\right)\right)+\pi \right)\times \exp\left(-\left|\frac{f\left(x_{a2}\right)-f\left(x_{a3}\right)}{\omega }\right|\right)\\ \omega =\max\left(f\left(x_{a1}\right),f\left(x_{a2}\right),f\left(x_{a3}\right)\right) \end{array}\right.$$ (21)

where *f(x)* is the value of the objective function, $WM2_{l}^{g}$ is defined as


$$WM2_{l}^{g}=\delta \times \frac{w_{1}\left(x_{bs}-x_{bt}\right)+w_{2}\left(x_{bs}-x_{ws}\right)+w_{3}\left(x_{bt}-x_{ws}\right)}{w_{1}+w_{2}+w_{3}+\varepsilon }+\varepsilon \times rand,l=1,2,...,n$$ (22)

where the weighting functions are as follows.


$$\left\{ \begin{array}{c} w_{1}=\cos\left(\left(f\left(x_{bs}\right)-f\left(x_{bt}\right)\right)+\pi \right)\times \exp\left(-\left|\frac{f\left(x_{bs}\right)-f\left(x_{bt}\right)}{\omega }\right|\right)\\ w_{2}=\cos\left(\left(f\left(x_{bs}\right)-f\left(x_{ws}\right)\right)+\pi \right)\times \exp\left(-\left|\frac{f\left(x_{bs}\right)-f\left(x_{ws}\right)}{\omega }\right|\right)\\ w_{3}=\cos\left(\left(f\left(x_{bt}\right)-f\left(x_{ws}\right)\right)+\pi \right)\times \exp\left(-\left|\frac{f\left(x_{bt}\right)-f\left(x_{ws}\right)}{\omega }\right|\right)\\ \omega =\max\left(f\left(x_{ws}\right)\right) \end{array}\right.$$ (23)

where *x*_*bs*_, *x*_*bt*_ and *x*_*ws*_ three vectors represent the best, better and worst solutions in the *g*^*th*^ generation, respectively.

The optimization algorithm requires global search. The main definition of the INFO update rule is as follows


$$z1_{l}^{g}=\left\{ \begin{array}{c} x_{l}^{g}+\sigma \times MeanRule+rand\times \frac{\left(x_{bs}-x_{a1}^{g}\right)}{\left(f\left(x_{bs}\right)-f\left(x_{a1}^{g}\right)+1\right)},r<0.5\\ x_{a}^{g}+\sigma \times MeanRule+rand\times \frac{\left(x_{a2}^{g}-x_{a3}^{g}\right)}{\left(f\left(x_{a2}^{g}\right)-f\left(x_{a3}^{g}\right)+1\right)},r\geq 0.5 \end{array}\right.$$ (24)

$$z2_{l}^{g}=\left\{ \begin{array}{c} x_{bs}+\sigma \times MeanRule+rand\times \frac{\left(x_{a1}^{g}-x_{b}^{g}\right)}{\left(f\left(x_{a1}^{g}\right)-f\left(x_{a2}^{g}\right)+1\right)},r<0.5\\ x_{bt}+\sigma \times MeanRule+rand\times \frac{\left(x_{a1}^{g}-x_{a2}^{g}\right)}{\left(f\left(x_{a1}^{g}\right)-f\left(x_{a2}^{g}\right)+1\right)},r\geq 0.5 \end{array}\right.$$ (25)

where $z1_{l}^{g}$ and $z2_{l}^{g}$ are new vectors of *g*^*th*^ generation. σ is the contraction rate of the vector, which can be defined as follow


σ=2×α×rand−α (26)

The convergence acceleration part helps the algorithm speed up convergence by randomly selecting an excellent vector and combining it with the current vector to drive the population towards the global optimal solution.

(2) Vector combining stage

The method integrates the two vectors produced in the preceding stage with the current vector to improve local search efficacy and population variety. The merge operation is based on a certain probability, and if the conditions are met, the new vector is combined with the current vector to form a new vector.


$$u_{l}^{g}=\left\{ \begin{array}{c} z1_{l}^{g}+\mu \cdot \left|z1_{l}^{g}-z2_{l}^{g}\right|,r_{1}<0.5\&\&r_{2}<0.5\\ z1_{2}^{g}+\mu \cdot \left|z1_{l}^{g}-z2_{l}^{g}\right|,r_{1}<0.5\&\&r_{2}\geq 0.5\\ x_{l}^{g},r_{1}>0.5 \end{array}\right.$$ (27)

where $u_{l}^{g}$ is the obtained vector using the vector combining in *g*^*th*^ generation, *μ* is equal to 0.05 × *rand*.

(3) Local search stage

The primary objective of the local search phase is to enhance the convergence rate and prevent the algorithm from becoming ensnared in local optima, hence facilitating a more effective exploration of the global optimum. This stage uses globally optimal positions and mean-based rules to produce a new vector that will be used to improve the current solution. The vector definition formula is as follows:


$$u_{l}^{g}=\left\{ \begin{array}{c} x_{bs}+rand\times \left(MeanRule+rand\times \left(x_{bs}^{g}-x_{a1}^{g}\right)\right),r_{1}<0.5\&\&r_{2}<0.5\\ x_{rnd}+rand\times \left(MeanRule+rand\times \left(h_{2}*x_{bs}-h_{1}*x_{rnd}\right)\right),r_{1}<0.5\&\&r_{2}\geq 0.5 \end{array}\right.$$ (28)

where *x*_*rnd*_ is a solution consisting of *x*_*avg*_, *x*_*bt*_, and *x*_*bs*_.


$$x_{rnd}=\varphi *x_{avg}+\left(1-\varphi \right)*\left(\varphi *x_{bt}+\left(1-\varphi \right)*x_{bs}\right)$$ (29)

$$x_{avg}=\left(x_{a}+x_{b}+x_{c}\right)/3$$ (30)

where *φ* is a random number between [0, 1], *h*_*1*_ and *h*_*2*_ are random numbers defined as


$$h_{1}=\left\{ \begin{array}{c} 2\times rand,k>0.5\\ 1,k\geq 0.5 \end{array}\right.$$ (31)

$$h_{2}=\left\{ \begin{array}{c} rand,k>0.5\\ 1,k\geq 0.5 \end{array}\right.$$ (32)

**Fig 11:**
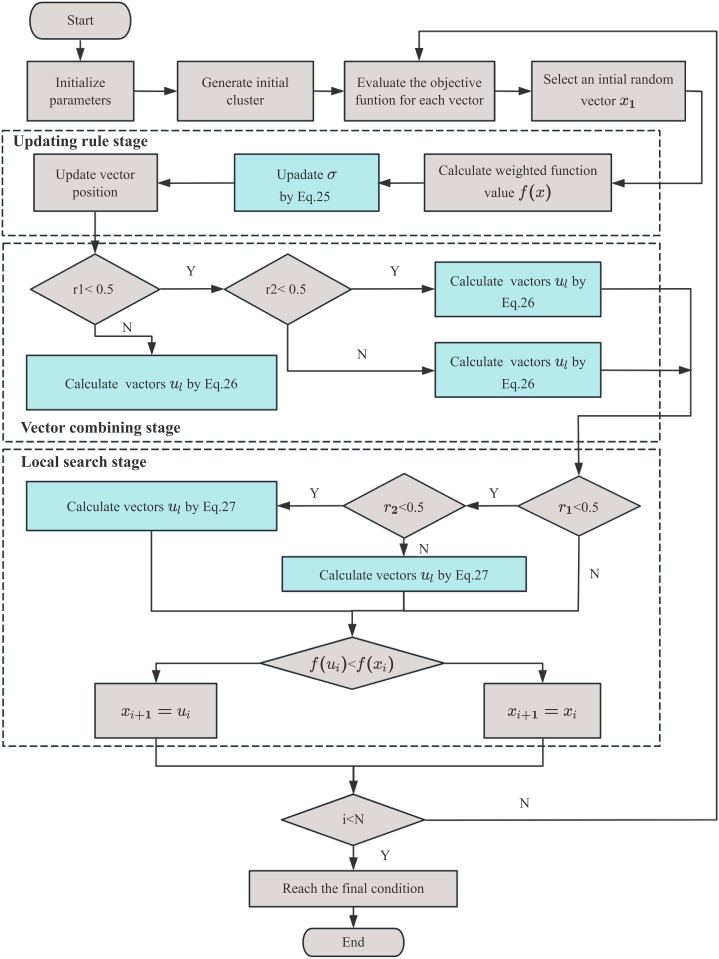
Flow chart of INFO algorithm.

The INFO algorithm will persist in its iterations until a specified termination criterion is met, which may involve achieving a maximum iteration threshold or a solution quality index that satisfies a defined optimization standard, thereby ensuring the acquisition of satisfactory results within reasonable computational resources. After each iteration, the algorithm updates the global optimal solution and continues to search for a better one. The flowchart of the INFO for parameter optimize of the HMMS is shown in Fig 11.

As shown in Table 3, the electromagnetic performance of the proposed HMMS has been compared with that of five other MS. It can be observed that the proposed scheme achieves the highest thrust density and the lowest fluctuation through the integration of an asymmetric Halbach topology and infographic-based KELM optimization. The implementation of the asymmetric Halbach structure results in a peak magnetic flux density of 1.16 T, representing an improvement of 3.6–31.8% over existing schemes. The asymmetric magnetization angle enhances the magnetic field focusing effect, while discretized segmentation effectively mitigates the leakage magnetic field. The thrust of the proposed scheme reaches 4.96 kN, marking a 40.8% increase compared to the initial design model. Additionally, the THD of the proposed scheme is as low as 1.57%, which is 21.4% to 74.3% lower than those reported in the literature [12,15,23]. This low fluctuation is attributed to the reduction in the sinusoidal distortion rate of the magnetic flux density waveform from 6.12% to 4.55%, along with the suppression of non-working harmonics in the optimized harmonic spectrum. Although the theoretical magnetic flux density of the continuous helical Halbach structure in references [14,19] reaches 1.12 T and 1.05 T, respectively, only simulation results are provided without prototype verification.

**Table 3:** Performance comparison of the existing HMMSs and the proposed HMMS.

Study Source	Optimization Method	Magnetization Topology	Airgap Flux Density (T)	Thrust Force (kN)	Thrust Improve-ment	Thrust Ripple (THD)	Validation Method
HMMS [12]	Gradient Descent	α-Gradient Halbach	0.88	2.95	+12%	**2.10%**	Platform Test
HMMS [14]	NSGA-II	Composite Rotor Halbach	1.12	4.37	+35%	2.40%	Scaled Model Test
HMMS [15]	Experimental Trial	Discrete Halbach (3-segment)	0.92	3.21	+15%	5.20%	Platform Test
HMMS [19]	Analytical Model	Continuous Helical Halbach	1.05	3.89	+28%	3.80%	FEA Verification
HMMS [23]	Parametric Sweep	Symmetric Halbach (4-segment)	0.98	3.52	+22%	4.15%	FEA Verification
**Proposed**	**INFO-KELM**	**Asymmetric Halbach (2 and 3 segments)**	**1.16**	**4.96**	**+40.8%**	**1.57%**	**Platform Test**

#### 3.3.3. INFO-KELM model.

The effectiveness of the KELM is substantially affected by model parameters, such as the regularization coefficient *C* and the kernel function parameter *k*. The INFO algorithm is applied to KELM parameter optimization, and the INFO-KELM parameter optimization model is established to improve the optimization calculation accuracy.

The specific steps to optimize modeling based on INFO_DELM are:

(1) Divide and normalize the data set, and preprocess the original output data to reduce the impact of variable differences on the model.
(2) Initialize KELM network parameters, and randomly initialize KELM regularization coefficients and kernel function parameters.
(3) Optimize KELM parameters based on INFO, initialize the population, and update, merge and search the selected vectors.
(4) Ascertain if a combination of parameters exists that can enhance the model’s predictive efficacy. If not, revert to Step 3 to proceed with the iterative computation.
(5) The input data is processed by the hidden layer neuron to provide the hidden layer output, which is then linearly mixed with the output weight to yield the optimization result.

**Fig 12:**
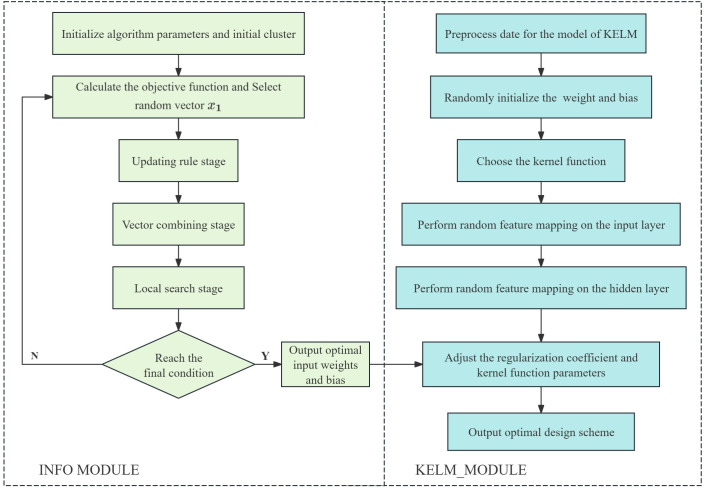
Flow chart of INFO-KELM model.

The modelling procedure of INFO-KELM is illustrated in Fig 12.

**Fig 13:**
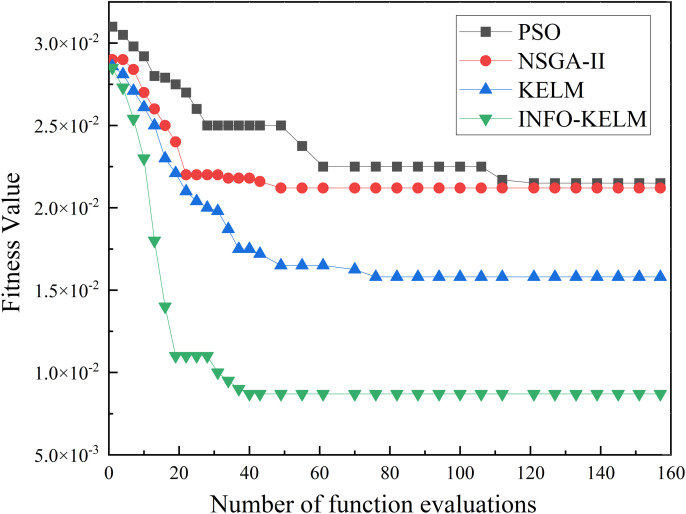
The convergence curves of the HMMS optimization algorithm based on INFO-KELM and other algorithms.

Fig 13 illustrates the convergence curves of INFO-KELM and the other three algorithms with respect to the number of iterations. INFO-KELM demonstrates a tendency to converge to optimality within approximately 40 iterations and achieves the lowest final fitness value. This indicates that both its search efficiency and accuracy surpass those of the other methods. Although KELM also exhibited rapid fitness reduction in the initial stage, it quickly plateaued and remained at a relatively high fitness level, suggesting that despite its fast convergence speed, it converges to a suboptimal solution. The descent curve of Non-Dominated Sorting Genetic Algorithm II (NSGA-II) is relatively gradual, requiring more iterations to approach the optimal region, thus indicating a moderate convergence speed. Particle Swarm Optimization (PSO) exhibits the slowest convergence speed and achieves the lowest final accuracy.

**Fig 14:**
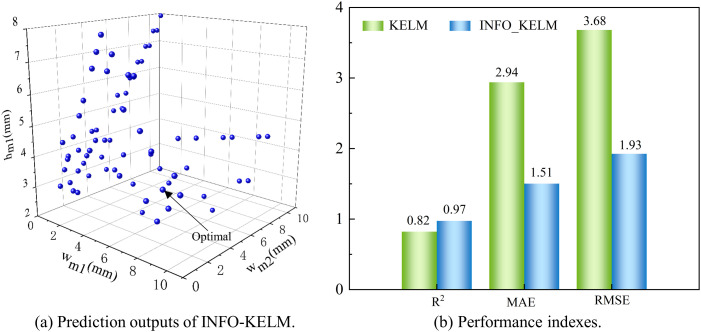
INFO-KELM optimization results.

The optimization based on INFO-KELM Fig 14a takes three parameters as an example to show a set of solution sets obtained by the INFO-KELM optimization algorithm. Through the comparison of multiple groups of result parameters and comprehensive consideration of manufacturing processing size requirements, a set of optimal solutions can be selected. Based on the principles of INFO algorithm and KELM, the INFO-KELM model is established. The maximum number of generations permitted for iteration is established at 30, and each layer has 50 hidden nodes. The prediction accuracy of KELM is typically not expressed by a single parameter but evaluated using a series of performance metrics. The coefficient of determination (*R*^*2*^), the mean absolute error (MAE), and the root mean square error (RMSE) are the metrics that are utilized for the purpose of evaluating the model. A higher *R*^*2*^ value, nearer 1, denotes a more accurate forecast. RMSE provides an error measure in the same units as the original data, facilitating the visualization of prediction error magnitude. MAE, being insensitive to outliers due to its reliance on absolute error values, offers a robust measure of prediction accuracy. Lower RMSE and MAE values signify superior performance.

The performance of the INFO-KELM model is assessed by comparing it with the regular KELM model, with the findings illustrated in Fig 14b. The *R*^*2*^ value of the INFO-KELM model exceeds that of the KELM model by 0.15, signifying a notable increase in accuracy. Furthermore, the MAE and RMSE metrics of the INFO-KELM model are inferior to those of the KELM model, indicating superior stability and dependability.

Based on the INFO-KELM optimization design and actual processing conditions, the optimal structural parameters of the HMMS can be obtained, and the results are presented in Table 4. Owing to the constraints of manufacturing and processing technologies, a configuration of solutions with integer structural parameters is chosen as the definitive optimized structure.

**Table 4:** Optimization results of structural parameters.

Parameters	Definitions	Units	Values
$R_{1}$	Outer radius of translator	mm	19
$R_{2}$	Inner radius of rotor	mm	31
$w_{m1}$	Width of radial translator PMs	mm	5
$h_{m1}$	Thickness of translator PMs	mm	6
$w_{m2}$	Width of high rotor PMs	mm	4
$h_{m2}$	Thickness of high rotor PMs	mm	6
$h_{m3}$	Thickness of low rotor PMs	mm	5
$w_{b}$	Width of bulge	mm	0.5
$h_{b}$	Thickness of bulge	mm	0.5

## 4. Results and discussion

Based on the multi-objective optimization results of INFO-KELM, this section quantifies the electromagnetic performance improvement of HMMS through three-dimensional FEA. The optimized HMMS is verified in two key aspects, quantifying the electromagnetic performance via three-dimensional transient finite element analysis and harmonic distortion analysis using FFT.

**Fig 15:**
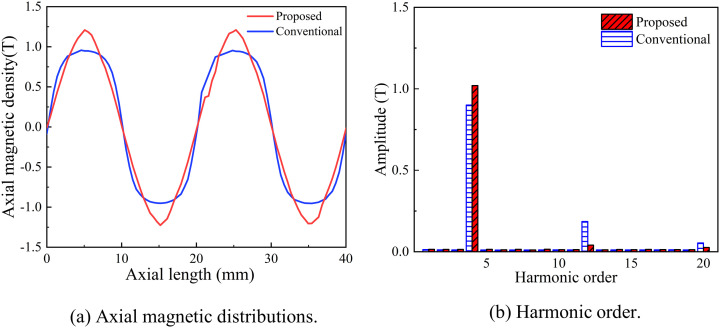
Comparison of axial magnetic density before/after optimization.

As illustrated in Fig 15a, by utilizing an improved Halbach PM array combined with optimized size parameters, the Halbach array significantly enhanced the magnetic field intensity on the air gap side. The amplitude of the radial flux density increases, with the peak value rising from 0.96 T to 1.16 T. Additionally, the sinusoidal distortion of the magnetic density waveform decreased from 6.12% to 4.55%, resulting in a more sinusoidal waveform. Effective working harmonics are notably amplified, while non-working harmonics are effectively suppressed. Specifically, the fourth harmonic increased from 0.89 T to 1.02 T, representing a 15.6% improvement, as shown in Fig 15b.

**Fig 16:**
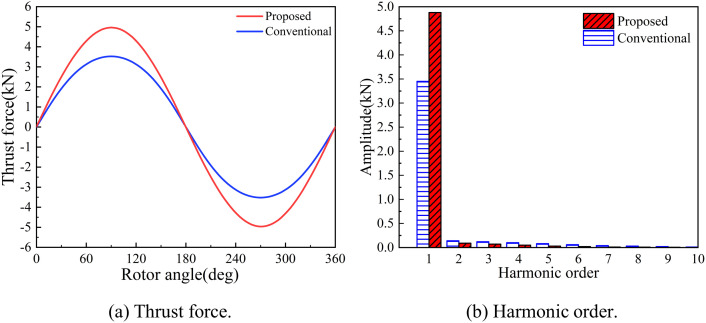
Comparison of thrust force before/after optimization.

As illustrated in Fig 16a, the peak thrust value of the optimized HMMS model increased from 3.52 kN to 4.96 kN, representing a 40.8% improvement. The fast fourier transform (FFT) harmonic analysis of the thrust waveform is shown in Fig 16b. It can be observed that after optimization, the fundamental amplitude of the thrust significantly increases, while the content of the remaining harmonics decreases, and the harmonic distortion rate of the waveform reduces from 4.16% to 1.57%. Consequently, the thrust performance of the proposed HMMS, optimized using the INFO-KELM model, has been substantially enhanced.

## 5. Exprimental verification

**Fig 17:**
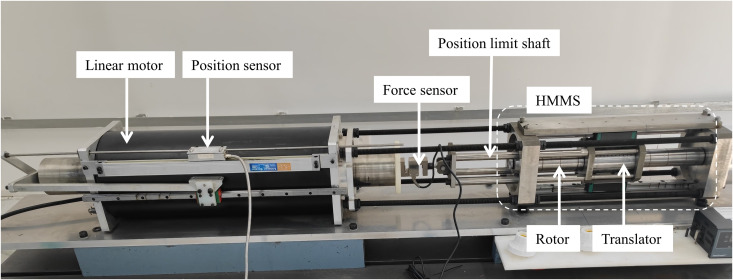
Laboratory test platform for full-size HMMS prototypes.

To assess the validity of the theoretical analysis and finite element simulation results for the proposed HMMS, a prototype is constructed, its thrust characteristics are measured, and a testing platform is established, as shown in Fig 17.

The HMMS prototype translator travels linearly along the z-axis, while the rotor revolves around the same axis. The PM is made of NdFeB38 rare earth material, with steel A3 serving as both the translator and rotor back irons. The exterior of the screw is encased in a slender stainless steel sleeve to safeguard the PM from environmental factors. The control system consists of a host computer and a motion controller, which accurately regulate the speed and displacement of the driving motor.

**Fig 18:**
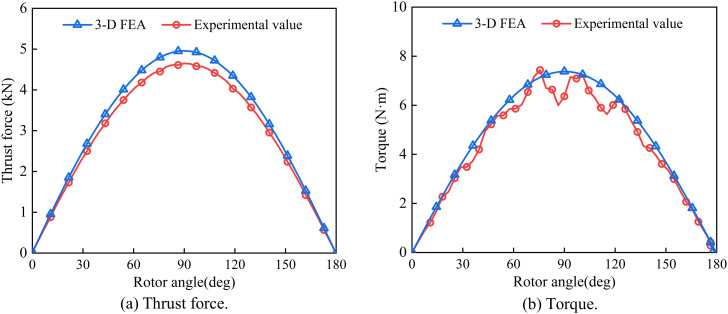
Comparison of 3-D FEA and experimental waveforms.

The waveforms of thrust force and torque are depicted in Fig 18. The maximum thrust obtained from the three-dimensional finite element simulation was 4.96kN, while the observed peak was 4.59kN, which decreased by 8.1% compared with the simulation results. This difference can be attributed to processing errors and the non-uniform fitting of helical magnets. The positive peak torque value is 7.45 N·m, confirming that the magnetic screw generates two equal-amplitude reverse electromagnetic thrust forces within one mechanical pitch, which aligns with the bipolar magnetic field distribution. The experimental torque curve between 60° and 120° exhibits a slight sawtooth pattern, with relatively large high-order harmonic amplitudes. This is due to the local saturation and air gap magnetic field fluctuations caused by the mechanical gap and assembly errors existing between the HMMS PMs. Overall, the calculated results demonstrate good agreement with the measured results in terms of thrust and torque amplitudes, as well as pulsation periods.

**Fig 19:**
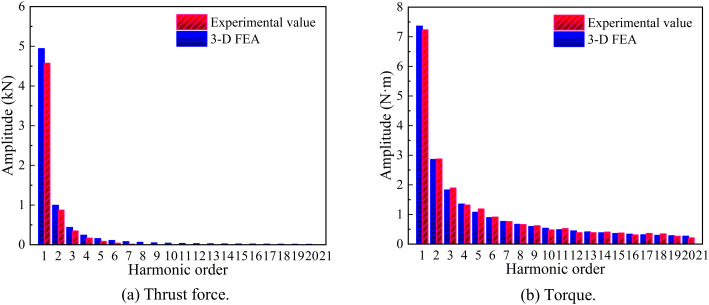
Comparison of 3-D FEA and experimental harmonics.

To quantitatively evaluate the distortion levels of thrust and torque, a comparative harmonic analysis between experimental and simulation results was conducted, as illustrated in Fig 19. The fundamental harmonic amplitudes in both scenarios slightly exceeded their experimental counterparts, while the mean output of experimental data demonstrated satisfactory agreement with simulation data. For the HMMS, the 2nd and 4th harmonic orders directly contributed to torque pulsations, potentially inducing mechanical vibrations and axial impacts. As evidenced in Fig 19b, harmonic components from the 6th to 21st orders accounted for approximately 48% of the fundamental wave energy, contrasting sharply with the mere 7% observed in Fig 19a. The total harmonic distortion (THD) values exhibited remarkable consistency across all orders.

## 6. Conclusion

This study proposes and validates an integrated design framework that combines a helical- HMMS with an INFO-KELM surrogate model to enhance the efficiency of WECs. The following is a summary of the key contributions of this study.

1) A sensitivity-guided, surrogate-assisted multi-objective optimization strategy that leverages INFO-KELM for rapid, high-fidelity performance prediction.
2) An advanced helical Halbach topology that effectively concentrates the air-gap flux, minimizes harmonic distortion, and achieves a 40.8% increase in peak thrust compared to a radially magnetized baseline when coupled with the optimization framework.
3) Experimental verification was conducted using a full-scale prototype. The results demonstrated that the deviation between the predicted thrust force from the optimized simulation data and the experimental measurements was less than 8.1%, thereby validating the effectiveness and accuracy of the proposed method.

Future work will focus on reducing the lightweight costs at small scales, conducting long-term trials under real sea conditions, and extending the application of INFO-KELM to other types of WECs.

## Supporting information

S1 File Table 1. Sensitivity analysis. Table 2. Comparison of 3-D FEA and experimental value. (DOCX)
