# Supplementary material for: Optimal design of Halbach magnetized magnetic screw for wave energy converters based on KELM network optimized by weighted mean of vectors algorithm
Source: PLoS One. 2025 Aug 14;20(8):e0329295. doi: 10.1371/journal.pone.0329295 (PMC12352764; doi:10.1371/journal.pone.0329295)
Supplement: S1 File — (DOCX) [file pone.0329295.s001.docx]

**Minimal Data Set**

1. Sensitivity analysis.

| **Parameters** | ***S_Fmax_(x_i_)*** | ***S_Bz_(x_i_)*** | ***S_kB_(x_i_)*** |
| --- | --- | --- | --- |
| *R_1_* | 0.095 | 0.075 | 0.301 |
| *R_2_* | 0.023 | 0.135 | 0.043 |
| *w_m1_* | 0.416 | 0.369 | 0.471 |
| *w_m2_* | 0.408 | 0.378 | 0.469 |
| *h_m1_* | 0.585 | 0.401 | -0.196 |
| *h_m2_* | 0.556 | 0.388 | -0.217 |
| *h_m3_* | 0.089 | 0.027 | 0.115 |
| *w_b_* | -0.101 | -0.075 | 0.022 |
| *h_b_* | -0.095 | 0.301 | 0.076 |

1. Comparison of 3-D FEA and experimental value.

| **Rotor angle (deg)** | **Thrust force (kN)** | | **Torque (N·m)** | |
| --- | --- | --- | --- | --- |
|  | **3-D FEA** | **experimental value** | **3-D FEA** | **experimental value** |
| 0 | 0 | 0 | 0 | 0 |
| 10 | 0.96 | 0.88 | 1.41 | 1.22 |
| 20 | 1.83 | 1.71 | 2.74 | 2.47 |
| 30 | 2.48 | 2.31 | 3.92 | 3.41 |
| 40 | 3.16 | 2.96 | 4.71 | 4.19 |
| 50 | 3.81 | 3.57 | 5.67 | 5.57 |
| 60 | 4.25 | 3.98 | 6.45 | 5.86 |
| 70 | 4.58 | 4.30 | 6.81 | 6.95 |
| 80 | 4.86 | 4.56 | 7.21 | 6.69 |
| 90 | 4.96 | 4.65 | 7.37 | 6.36 |
| 100 | 4.87 | 4.57 | 7.24 | 7.23 |
| 110 | 4.56 | 4.28 | 6.82 | 5.93 |
| 120 | 4.09 | 3.85 | 6.11 | 6.27 |
| 130 | 3.82 | 3.57 | 5.68 | 5.34 |
| 140 | 3.16 | 2.95 | 4.69 | 4.26 |
| 150 | 2.36 | 2.21 | 3.51 | 3.32 |
| 160 | 1.51 | 1.40 | 2.23 | 2.04 |
| 170 | 0.92 | 0.86 | 1.35 | 1.25 |
| 180 | 0.01 | 0.01 | 0.01 | 0.01 |
